# Chronic Neck Pain and Cervico-Craniofacial Pain Patients Express Similar Levels of Neck Pain-Related Disability, Pain Catastrophizing, and Cervical Range of Motion

**DOI:** 10.1155/2016/7296032

**Published:** 2016-03-29

**Authors:** Daniel Muñoz-García, Alfonso Gil-Martínez, Almudena López-López, Ibai Lopez-de-Uralde-Villanueva, Roy La Touche, Josué Fernández-Carnero

**Affiliations:** ^1^Department of Physiotherapy, Faculty of Health Science, Centro Superior de Estudios Universitarios La Salle, Universidad Autónoma de Madrid, 28023 Madrid, Spain; ^2^Research Group on Movement and Behavioural Science and Study of Pain, Centro Superior de Estudios Universitarios La Salle, Universidad Autónoma de Madrid, 28023 Madrid, Spain; ^3^Institute of Neuroscience and Craniofacial Pain (INDCRAN), 28023 Madrid, Spain; ^4^Hospital La Paz Institute for Health Research, IdiPAZ, 28046 Madrid, Spain; ^5^Department of Psychology, Universidad Rey Juan Carlos Alcorcón, 28922 Madrid, Spain; ^6^Department of Physical Therapy, Occupational Therapy, Rehabilitation and Physical Medicine, Universidad Rey Juan Carlos, 28922 Madrid, Spain

## Abstract

*Background.* Neck pain (NP) is strongly associated with cervico-craniofacial pain (CCFP). The primary aim of the present study was to compare the neck pain-related disability, pain catastrophizing, and cervical and mandibular ROM between patients with chronic mechanical NP and patients with CCFP, as well as asymptomatic subjects.* Methods.* A total of 64 participants formed three groups. All participants underwent a clinical examination evaluating the cervical range of motion and maximum mouth opening, neck disability index (NDI), and psychological factor of Pain Catastrophizing Scale (PCS).* Results.* There were no statistically significant differences between patients with NP and CCFP for NDI and PCS (*P* > 0.05). One- way ANOVA revealed significant differences for all ROM measurements. The post hoc analysis showed no statistically significant differences in cervical extension and rotation between the two patient groups (*P* > 0.05). The Pearson correlation analysis shows a moderate positive association between NDI and the PCS for the group of patients with NP and CCFP.* Conclusion.* The CCFP and NP patient groups have similar neck disability levels and limitation in cervical ROM in extension and rotation. Both groups had positively correlated the NDI with the PCS.

## 1. Introduction

Neck pain (NP) is common in the adult general population, with prevalence estimates of between 30 and 50% [1] showing an incidence rate between 146 and 213 per 1,000 patients per year. Furthermore, cervical disorders have been recognized as the possible cause of pain and disorders in distant structures [2]. Nearly half of NP patients develop chronic symptoms [3] and many will continue to exhibit moderate disability at long-term follow-up [4]. The economic burden associated with the management of NP is second only to low-back pain in annual workers compensation costs in the United States [5]. Although the etiology of insidious mechanical NP is under debate, it is clear that NP is multifactorial in nature, with both physical and psychosocial contributors [6, 7].

Many NP patients will have disability at long-term follow-up, which may lead in physical and psychosocial contributors [8].

Recent research has shown that neck pain-related disability alters the normal function of craniomandibular region [9, 10]. Furthermore, several epidemiological studies have reported that patients with NP often report pain in different conditions involving the temporomandibular joint (TMJ) and the craniofacial region [11–16]. The risk of being diagnosed with pain in both regions is higher in women than men [17]. The interaction demonstrated by the literature between neck pain and craniofacial pain [18–20] has led some researchers to consider the word “cervico-craniofacial pain” (CCFP) to integrate these conditions. The CCFP is defined as “the presence of mechanical signs of dysfunction and muscular pain (e.g., limited movement, uncoordinated movement, and weakness and lack of endurance in the neck and jaw) that were exacerbated by movement and maintained postures, and that generates pain in the cervical and craniofacial regions” [21, 22].

Moreover, it appears that an intimate functional relationship exists between the neck and the craniofacial region as suggested by their anatomical and biomechanical interrelationships [15]. The neuroanatomical basis for the relationship between the head and neck may be related to the trigeminocervical nucleus caudalis in the spinal grey matter of the spinal cord at the C1–C3 level, where there is a convergence on the nociceptive second-order neurons receiving trigeminal and cervical inputs [23]. The topographic arrangement of the trigeminal nucleus caudalis allows the interchange of nociceptive information between the cervical spine and the trigeminal nerve [24]. Several studies have demonstrated that stimulation of trigeminal-innervated structures evoked painful sensations in the neck and vice versa [25, 26]. In addition, it has been reported that injection of an inflammatory irritant into deep paraspinal tissues results in a sustained activation of both jaw and neck muscles [27, 28].

Furthermore, craniofacial pain is associated with myofascial pain and can lead to a psychophysiological disorder [29] involving central nervous system pain-regulatory systems which results in physiological and neuroendocrine responses to emotional and physical stressors. In addition, many studies have established that a substantial proportion of patients with CCFP also suffer from psychological disorders [30]. A recent cohort study found that catastrophizing contributed to the intensity and disability of pain and the progression of the disorders [31]. Other authors also reported that higher levels of catastrophizing decrease the chance of treatment success in patients [32]. Given previous evidence, this study hypothesized that patients with CCFP have higher levels of neck disability and pain catastrophizing and reduced range of motion (ROM) than patients with NP and asymptomatic subjects.

We consider that patients presenting both conditions (craniofacial pain and neck pain) could alter cervical function and produce higher levels of disability and lower ranges of cervical motion.

Therefore, the primary aim of the present study was to compare the neck pain-related disability, pain catastrophizing, and cervical and mandibular ROM between patients with chronic mechanical NP and patients with CCFP, as well as asymptomatic subjects. The secondary objective is to determine the association between neck pain-related disability and pain catastrophizing with ranges of motion in patients with NP and CCFP.

## 2. Methods

A cross-sectional study was developed. The study was conducted as single-blind (where the evaluator did not know the patients' conditions) study and following international recommendations for the strengthening and the reporting of observational studies in epidemiology (STROBE) [33]. The protocol was approved by the local Ethics Committee (CSEULS-PI-004/2013) and conducted following the Helsinki Declaration.

### 2.1. Subjects

A consecutive nonprobabilistic convenience sample of patients with NP, CCFP, and asymptomatic subjects for the control group were recruited between January 2014 and October 2015. The sample was recruited from our university campus and the local community through flyers, posters, and social media in Madrid (Spain) and they were between 18 and 65 years old. An 11-point NRS (0 = no pain, 10 = maximum pain) was used to assess pain levels [34].

Patients presenting with chronic NP and patients diagnosed with CCFP were screened for eligibility criteria if they presented at least a pain rating of 3 on NPRS during the last 3 months.

Patients for the NP group were included with symptoms isolated to the neck region with cervical mechanical pain (neck and/or shoulder pain with symptoms provoked by neck postures, neck movement, or palpation of the cervical musculature) following the recognition of cervical spinal pain based on the procedures described by Visscher et al. (2000) [35] and a Neck Disability Index (NDI) score of at least 5 [36]. Patients were included for the CCFP group if they have the inclusion criteria for the NP group and also they were a primary diagnosis of myofascial pain following Axis 1 (myofascial pain) of the Research Diagnostic Criteria for Temporomandibular Disorders (TMD) [37]; and bilateral pain of the temporal, masseter, suboccipital, and trapezius muscles.

Patients were excluded for any of the following: previous surgery for treatment of TMD pain; history of rheumatoid disease; extensive anatomical destruction or deterioration of the TMJ; being diagnosed as having pain of neuropathic or odontogenic origin; having a diagnosis of psychosis; current use of antidepressants or anxiolytics; taking narcotic pain medication; or pregnancy (due to prescription of nonsteroidal anti-inflammatory drugs).

Finally, asymptomatic controls were recruited from volunteers who responded to a local announcement and were excluded if they exhibited a history of neck, facial, or head pain (infrequent episodic tension-type headache was permitted), or any systemic disease or diagnosis compatible with the inclusion criteria.

## 3. Procedure

The study protocol was the same for the NP and CCFP patients and the asymptomatic control group, who all signed a consent form before participation. All examinations were done in a quiet, draught-free, temperature- and humidity-controlled laboratory (24°C ± 1°C, relative humidity 25–35%). This protocol was performed by an evaluator who had not participated in the selection and data collection procedures, to ensure blinding status of the investigation.

After consenting to the study, the recruited patients were given a battery of questionnaires to complete on the day of the evaluation. These included various self-reports for demographic data and pain-related disability variables. The sociodemographic questionnaire collected information about the following variables: age, gender, and duration of pain. Measures of pain catastrophizing were assessed using the Pain Catastrophizing Scale (PCS); neck pain-related disability was collected using the Spanish version of NDI [36]. Once patients completed all self-reports, the research proceeded to evaluate the cervical ROM and maximal mouth opening (MMO).

### 3.1. Primary Outcomes

NDI was used to measure perceived disability. The NDI consisted of 10 questions measured on a 6-point scale (0 = no disability, 5 = full disability). The NDI has been demonstrated to be a reliable (intraclass correlation coefficients ranging from 0.50 to 0.98) and valid self-assessment for disability in chronic NP patients [36].

Pain Catastrophizing. To evaluate participants' propensity to catastrophize about pain we used the Spanish version [38] of the Pain Catastrophizing Scale (PCS). This scale is a 13-item questionnaire designed to measure the three components of catastrophizing: rumination, magnification, and helplessness.

### 3.2. Secondary Outcomes

The ROM of the neck was measured with the cervical ROM (CROM). This device consists of a plastic frame that rests on the nose and ears and is attached to the head via a Velcro strap. Three angles gauges were attached to the frame and arranged perpendicular to each other, indicating the range of cervical movement of the subject. Flexion, extension, and lateral flexion of the neck were recorded with gravity goniometers. Cervical rotation was measured with a goniometer compass that works in conjunction with a magnetic magnet placed around the neck [39]. Validity [40] and reliability have been demonstrated (both as intrarater and interrater [41]), to measure cervical ROM.

Patient was seated in a chair with back to 90 degrees, with feet flat on the floor, arms along the body, and head in neutral position. Verbal commands were given to the subjects to perform the neck movements until the pain or maximal ROM. First, assess mobility in the sagittal plane, followed by the frontal plane, and finally the transverse plane. The mean of 3 trials (intraexaminer reliability) was calculated and used for the analysis.

MMO was measured in millimetres with a digital calliper [42, 43]; a reduced opening capacity has been demonstrated in TMD patients relative to asymptomatic subjects. The head of the patient was controlled in a neutral position, since it has demonstrated the influence of the position in the measurement [44]. All measurements were made between the incisal edges of the upper and lower right central incisors. These measurements were made at least within 30 seconds to avoid wind-up phenomenon.

### 3.3. Statistical Analysis

All data analyses were performed on SPSS for Windows, Version 21.0 (SPSS Inc., Chicago, IL). The statistical analyses were conducted at a 95% confidence level and a *P* value less than 0.05 was considered statistically significant. Descriptive statistics was generated for the sociodemographic, psychological, and pain-related disability variables and physical measures. Results are expressed as mean, standard deviation (SD), with 95% confidence intervals (95% CI). A normal distribution of the data was confirmed with the Kolmogorov-Smirnoff test.

For comparison of the primary outcomes (NDI and PCS) between the two patients groups, a Student's *t*-test for independent samples was used. Effect sizes (Cohen's *d*) were calculated for outcome variables. According to Cohen's method, the magnitude of the effect was classified as small (0.20 to 0.49), medium (0.50 to 0.79), or large (≥0.8) [45].

One-way ANOVA was used to analyze the group factor for ROM variables (cervical ROM and MMO). Significant ANOVA findings were followed up with post hoc test using the Bonferroni correction. Partial eta-squared (*η* ^2^_*p*_) was calculated as a measure of effect size (strength of association) for each main effect and interaction in the ANOVAs, with 0.01–0.059 representing a small effect, 0.06–0.139 a medium effect, and >0.14 a large effect [46].

The relationship between physical measures (cervical ROM and MMO) and self-reports for pain-related disability and psychological measures was examined using Pearson correlation coefficients. A Pearson correlation coefficient greater than 0.60 indicated a strong correlation, a coefficient between 0.30 and 0.60 indicated a moderate correlation, and a coefficient below 0.30 indicated a low or very low correlation [47].

## 4. Results

The sample was composed of 44 patients (22 patients with NP; 20 patients with CCFP) and 22 asymptomatic controls. The patients presented a mean age of 26.22 ± 4.18 (mean ± SD) years (NP group: 25.55 ± 4.23 years; CCFP group: 26.90 ± 4.12 years) and a female percentage of 61.9% years (NP group: 50%; CCFP group: 75%), whereas these values for the asymptomatic controls were 24 ± 4.58 and 54.5%, respectively. Hence, demographic data of the groups were similar and comparable (*P* > 0.05). In addition, both patients groups showed similar outcomes for cervical pain duration with 144.27 ± 155.96 weeks in NP group and 118 ± 118.54 weeks in CCFP group. The orofacial pain duration observed in patients with CCFP was 86.55 ± 74.76 weeks.

### 4.1. Comparisons between Groups

There were no statistically significant differences between patients with NP and those with CCFP for NDI and PCS (*P* > 0.05) (Table 1).

One-way ANOVA revealed significant differences for all ROM measurements [extension (*F* = 9.270; *P* < 0.001; *η* ^2^_*p*_ = 0.23); lateroflexion (*F* = 4.076; *P* = 0.022; *η* ^2^_*p*_ = 0.11); rotation (*F* = 5.278; *P* = 0.008; *η* ^2^_*p*_ = 0.14); MMO (*F* = 15.821; *P* < 0.001; *η* ^2^_*p*_ = 0.34)] except for cervical flexion (*F* = 2.140; *P* = 0.126; *η* ^2^_*p*_ = 0.06). The post hoc analysis showed no statistically significant differences in cervical extension and rotation between the two patient groups (*P* > 0.05); however, differences in these variables were found with the control group showing a large effect size (*d* ≥ 0.8). The ROM of cervical lateral-flexion was lower in the group of CCFP in relation to the other two groups with a medium-large effect size (*d* = 0.52 to 0.87) (Table 1).

MMO measurement was lower in the CCFP group with respect to the data obtained in the control group and NP group and this difference was statistically significant with large effect size (*P* < 0.001; *d* > 0.8) (Table 1).

### 4.2. Correlations Analysis

The Pearson correlation analysis shows a moderate positive association between NDI and the PCS for the group of patients with NP and CCFP. These results are presented in Table 2.

Table 2 shows a significant negative moderate correlation between the ROMs in flexion (*r* = −0.452; *P* < 0.03) and lateroflexion (*r* = −0.507; *P* < 0.01), with PCS in patients with NP; the lower the ROM in flexion, right and left lateral flexion, and right rotation, the higher the PCS levels.

For patients with CCFP, a significant negative moderate correlation between the cervical ROM in rotation (*r* = −0.507; *P* < 0.04) with NDI is presented in Table 2.

## 5. Discussion

This survey has the main objective of comparing the data of disability, catastrophism, and ROM between the three groups studied.

It was observed that the NP and CCFP patients presented similar neck pain-related disability and pain catastrophizing levels and cervical ROM. Although these outcomes were not statistically significantly different, the lateroflexion ROM and MMO showed a medium effect size between the NP group and asymptomatic subjects with mean difference of the 95% CI included the minimal detectable change [48, 49]; therefore, this could be clinically relevant for some patients.

Moreover, both patient groups had less cervical ROM than asymptomatic subjects. MMO was lower in the CCFP and statistically significant differences were observed with respect to the other two groups. According to the results obtained in this investigation, the null hypothesis for comparing the variables neck pain-related disability, pain catastrophizing, and cervical ROM is assumed, between the NP and CCFP group and is rejected for the comparison of the cervical ROM variable between the CCFP group and the asymptomatic subjects.

### 5.1. Neck Disability and ROMs

We propose a hypothesis that presenting craniofacial pain and neck pain could generate greater levels of disability of neck and lower ranges of cervical motion than in patients who had neck pain only and viewing our results this was not found. Current evidence shows that the pain and disability of neck and jaw function alter the nociceptive processing at the trigeminal level [10] even when only neck pain [9, 50–52] occurs. However, our findings can not prove that this situation can be reversed. It is necessary to further investigate the possible influence of craniofacial cervical pain on motor activity, as well as disability and neck pain, as this issue is still unclear.

We found no correlations between cervical ROM and NDI in NP patients. This is in line with the findings of Cramer et al., who reported fair correlations in a large cohort study [53].

Our results for the CCFP group are consistent with Olivo et al. [54], who reported that patients with jaw pain had a high level of disability in the neck region. However, Olivo et al. reported less level of neck disability than identified here; they reported a score of 10.87 in patients with TMD, whereas we identified a mean score of 22.60 in patients with CCFP. This difference could be because the Olivo et al.'s study included myogenic or mixed TMD patients but not TMD patients suffering from neck pain. Most of the patients with NP and CCFP had moderate disability (63%), although there was more severe disability in patients with CCFP than NP (26.3% versus 18.2%).

Disability in patients with CCFP was found to be similar to patients with only NP. Although it has been suggested that disability scores of between 15 and 24 represent a moderate disability [55], other studies have suggested that a score of between 10 and 28 points in NDI represents mild disability [56]. Therefore, in our study the NP and CCFP patients showed a mild to moderate disability of the cervical spine. Although higher NDI scores in neck pain patients than reported here have been published, this could be because their patient populations had neck pain with and without symptoms to the upper extremity and also because they recruited patients from a hospital setting (i.e., where patients seek medical care) [57].

Cervical ROMs for both groups of our study were lower than typically reported values in angular ranges for similar age groups [41]. Also, the patients with NP and CCFP showed less cervical ROM and MMO than controls accounted for the largest proportions of variance in the self-report measures. However, it is likely that these measures alone are not sufficient to predict the disability status of patients with NP. Our finding of reduced ROM for the NP group is in agreement with several other studies [58]. A recent study of NP patients reported reduced ROM in extension for the upper cervical spine and reduced ROM in flexion for the lower cervical spine [59]; therefore, it is possible that the upper cervical spine is involved in nociceptive processing of CCFP. In connection with this, in recent research the presence of limitation of ROM in the upper cervical rotation mainly in TMD patients with headaches has been observed [60]. It is important to highlight that the CCFP group showed a statistically significant negative correlation between neck pain-related disability and the cervical rotation.

Various authors have studied the association between NP and craniofacial pain and their collected findings support the existence of a functional integration of the anatomic and biomechanical aspects of the craniomandibular and craniocervical regions [61]. Thus, the potential relationship between craniofacial pain and NP may be primarily expressed as the experiencing of pain in both locations. It should be noted that experimental studies showed the existence of neurologic circuits that allow convergence of proprioceptive and nociceptive afferences from C1–C4 to the trigeminal nucleus, as well as a close functional relationship between the cervical spine and the masticatory system [62]. Several studies have also reported a neuromuscular interaction between the jaw and neck muscles during functional contraction [63].

### 5.2. Disability and Psychological Associations

Our results support the previously proposed association between neck pain and temporomandibular disorders and emotional wellbeing and catastrophic thoughts [7, 27].

Our outcomes are also in agreement with other studies that have found that patients with TMD have higher levels of pain catastrophizing than pain-free subjects [64]. In addition, our results are consistent with previous research that has shown that catastrophizing is positively associated with pain intensity and disability in patients with TMD [31], as PCS scores correlated with several measures of cervical ROM.

The positive correlation between NDI and pain catastrophizing in NP and CCFP patients identified in our survey is in line with the findings of other authors that have identified an association between catastrophizing and disability, even when controlling for depression, anxiety, and pain severity [65].

The negative correlation between most of the cervical ranges of motion and PCS in our NP patient group is similar to literature findings that describe how pain-related catastrophizing plays an important role in the physical complaints of patients with chronic whiplash-associated disorders when referred to a physical therapist [66].

### 5.3. Clinical Implications

Currently, catastrophic behaviours to pain appear to be closely related to depressive symptoms and chronic pain. From a clinical point of view, it is essential to differentiate between patients not only based on physical examination but also on psychological factors. Our data show that patients with chronic NP or chronic CCFP could be involved in a similar trigeminal neurophysiology and exhibit similar levels of catastrophic thoughts. Therefore, such patients could benefit from similar treatments targeting the neck or craniofacial region. As catastrophizing is related to some disability measures, we also wonder whether it might be useful to complement physiotherapy treatment with psychological interventions.

## 6. Limitations

This study has some limitations. First, participants were recruited through flyers, posters, and social media, without them having requested health support. Differences between those that seek and those that do not seek health support have been reported, thus potentially introducing a further selection bias. This could have influenced some of our findings, for example, explaining why there was no difference between groups for pain catastrophizing. Second, limitation was not having included patients with craniofacial pain without neck symptoms. Third, although previous surveys similar to this used akin sample size, the results of the present study should be treated with caution because the sample size was small. Finally, we did not use a specific test to measure the craniofacial disability. Future studies resolving these limitations are needed.

## 7. Conclusion

The CCFP and NP patient groups have similar neck disability levels and limitation in cervical ROM in extension and rotation. Both groups had positively correlated the NDI with the PCS. Longitudinal research is needed to verify these associations.

## Figures and Tables

**Table 1:** Comparisons between groups.

Outcomes	Mean ± SD			Mean difference (95% CI); effect size (*d*)
	NP	CCFP	CG	(a) NP versus CCFP (b) NP versus CG (c) CCFP versus CG
NDI	21.82 ± 8.37	22.60 ± 8.24	—	(a) −0.78 (−5.97 to 4.41); *d* = −0.09

PCS	13.05 ± 10.27	17.45 ± 11.26	—	(a) −4.41 (−11.12 to 2.31); *d* = −0.40

Extension	65.48 ± 15.56	63.45 ± 17.29	80.91 ± 10.13	(a) 2.03 (−9.04 to 13.09); *d* = 0.12 (b) −15.43 (−26.23 to −4.63)^**∗∗**^; *d* = −1.17 (c) −17.46 (−28.53 to −6.39)^**∗∗**^; *d* = −1.23

Lateroflexion	84.93 ± 14.41	79.38 ± 15.99	91.96 ± 12.52	(a) 5.55 (−5.34 to 16.44); *d* = 0.36 (b) −7.02 (−17.65 to 3.61); *d* = −0.52 (c) −12.57 (−23.46 to −1.68)^**∗**^; *d* = −0.87

Rotation	129.52 ± 21.09	127.70 ± 17.63	143.82 ± 14.03	(a) 1.82 (−11.74 to 15.38); *d* = 0.09 (b) −14.30 (−27.53 to −1.06)^**∗**^; *d* = −0.80 (c) −16.12 (−29.68 to −2.56)^**∗**^; *d* = −1.01

MMO	47.51 ± 7.11	40.25 ± 7.49	52.35 ± 6.37	(a) 7.26 (1.95 to 12.58)^**∗∗**^; *d* = 0.99 (b) −4.83 (−10.02 to 0.35); *d* = −0.71 (c) −12.10 (−17.41 to −6.78)^**∗∗**^; *d* = −1.74

NDI, neck disability index; PCS, Pain Catastrophizing Scale; MMO, maximal mouth opening; NP, neck pain group; CCFP, cervico-craniofacial pain group; CG, control group.

^*∗*^ *P* < 0.05.

^*∗∗*^ *P* < 0.01.

**Table 2:** Correlations between patient groups.

	NDI		PCS	
	NP	CCFP	NP	CCFP
PCS	0.54^*∗∗*^	0.48^*∗*^	—	—
NDI	—	—	0.540^*∗∗*^	0.480^*∗*^
Flexion	−0.204	−0.382	−0.452^*∗*^	−0.219
Extension	−0.229	−0.145	−0.334	0.079
Lateroflexion	−0.327	−0.186	−0.507^*∗*^	0.168
Rotation	−0.256	−0.507^*∗*^	−0.386	−0.137
MMO	−0.117	0.194	0.122	−0.055

NDI, neck disability index; PCS, Pain Catastrophizing Scale; MMO, maximal mouth opening; NP, neck pain group; CCFP, cervico-craniofacial pain group.

^*∗*^ *P* < 0.05.

^*∗∗*^ *P* < 0.01.
